# P110 and P140 Cytadherence-Related Proteins Are Negative Effectors of Terminal Organelle Duplication in *Mycoplasma genitalium*

**DOI:** 10.1371/journal.pone.0007452

**Published:** 2009-10-14

**Authors:** Oscar Q. Pich, Raul Burgos, Enrique Querol, Jaume Piñol

**Affiliations:** Institut de Biotecnologia i Biomedicina and Departament de Bioquímica i Biologia Molecular, Universitat Autònoma de Barcelona, Barcelona, Spain; Baylor College of Medicine, United States of America

## Abstract

**Background:**

The terminal organelle is a complex structure involved in many aspects of the biology of mycoplasmas such as cell adherence, motility or cell division. *Mycoplasma genitalium* cells display a single terminal organelle and duplicate this structure prior to cytokinesis in a coordinated manner with the cell division process. Despite the significance of the terminal organelle in mycoplasma virulence, little is known about the mechanisms governing its duplication.

**Methodology/Principal Findings:**

In this study we describe the isolation of a mutant, named T192, with a transposon insertion close to the 3′ end of the *mg192* gene encoding for P110 adhesin. This mutant shows a truncated P110, low levels of P140 and P110 adhesins, a large number of non-motile cells and a high frequency of new terminal organelle formation. Further analyses revealed that the high rates of new terminal organelle formation in T192 cells are a direct consequence of the reduced levels of P110 and P140 rather than to the expression of a truncated P110. Consistently, the phenotype of the T192 mutant was successfully complemented by the reintroduction of the *mg192* WT allele which restored the levels of P110 and P140 to those of the WT strain. Quantification of DAPI-stained DNA also showed that the increase in the number of terminal organelles in T192 cells is not accompanied by a higher DNA content, indicating that terminal organelle duplication does not trigger DNA replication in mycoplasmas.

**Conclusions/Significance:**

Our results demonstrate the existence of a mechanism regulating terminal organelle duplication in *M. genitalium* and strongly suggest the implication of P110 and P140 adhesins in this mechanism.

## Introduction

Mycoplasmas are parasites or commensals of humans and other animals, characterized by the lack of a cell wall and fastidious nutritional requirements. Typically, these microorganisms with streamlined genomes are considered the smallest self-replicating cells known able to be grown in axenic culture. Consistent with this idea, the number of genes encoding proteins involved in cellular processes such as cell division, stress responses, and protein secretion, is certainly smaller in mycoplasmas as compared to other eubacteria. Similarly, many regulatory genes present in other bacteria, such as the two-component signal transduction systems, are missing in the majority of mycoplasmas. Nevertheless, mycoplasmas are furnished with sophisticated cytoskeletons made up of distinctive proteins [1], [2]. The cytoskeleton plays a key role in the biology of the mycoplasmas and provides the cells with a definite morphology. Interestingly, several *Mycoplasma* species have the ability to move along solid surfaces by means of intriguing mechanisms that are still poorly understood [3], [4], [5], [6], [7], [8].

*Mycoplasma genitalium* is one of the representatives of the *Mycoplasma pneumoniae* cluster as well as the mycoplasma with the smallest genome [9]. Actually, *M. genitalium* and *M. pneumoniae* show a similar chromosome organization and the average identity between their orthologs is 66.1% at the nucleotide level and 67.4% at the amino acid level [10]. A large number of the proteins comprising the *M. pneumoniae* cytoskeleton have been identified [11] and among them, we find a number of enzymes related with energy metabolism, lipoproteins and heat-shock proteins, as well as most of the cytadherence-related proteins. Characteristically, both *M. genitalium* and *M. pneumoniae* exhibit a specialized polar structure distinguished ultrastructurally by an electron-dense core that is a major constituent of the cytoskeleton [1], [12], [14]. This structure, also known as attachment organelle or terminal organelle (TO), mediates adherence to host target cells and is essential for gliding. Remarkably, the TO constitutes the actual gliding motor since isolated TOs recently detached from the cell body retain the ability to move [15] and also provides the guidance system to direct mycoplasma movement [16]. The machinery involved in mycoplasma gliding is becoming to be elucidated and we recently revaled a pivotal role of *mg200* and *mg386* genes in *M. genitalium* locomotion [17].

Mycoplasmas duplicate the TO prior to cytokinesis in a coordinated manner with the cell division process [18], [19]. Nascent TOs develop adjacent to a preexistent tip structure indicating that new TO formation is regulated spatially. Experiments performed on *M. pneumoniae* also show that mycoplasma cells become non-motile coincident with TO duplication. In a next step, the preexistent TO resumes gliding and provides the strength required to deliver the new TO to the opposite cell pole [20]. Seto and coworkers showed that chromosome replication is coincident with TO duplication [21]. Interestingly, the subcellular fraction containing the DNA replication complex of *M. gallisepticum* also contains the terminal tip structures, suggesting the association of the chromosomal DNA to the attachment organelle. Based on this observation, Quinlan and Maniloff proposed a role for the tip structure in the organization of DNA replication in this microorganism [22]. The likely association between the chromosome and specific components of the TO, supported by a recent report by Hatchel and Balish [6], would certainly facilitate a coordinated segregation of both structures. In agreement with this idea, we have proposed that MG218 protein, a key component of the *M. genitalium* electron-dense core, might also participate in the chromosome dynamics [13].

To date, four cytoskeletal proteins have shown to be necessary for TO development in *M. genitalium*: MG218, MG312, P140 and P110 [13], [23], [24]. MG218 and MG312, the orthologues of *M. pneumoniae* HMW2 and HMW1 proteins respectively, are thought to be structural components of the electron-dense core. P110, the orthologue of *M. pneumoniae* ORF6, is required for the stability of P140, the major cytadhesin of *M. genitalium* and the orthologue of *M. pneumoniae* P1. P140 and P110 are maintained at stoichiometric levels in *M. genitalium* reinforcing the close relationship existing between these two proteins [24]. The mechanisms regulating the duplication of the tip structure are much less understood. In a recent report, it has been shown that *M. pneumoniae* P24 seems to be necessary for new TO formation at WT rates [25]. However, no P24 ortologues have been currently identified in *M. genitalium* and the machinery implicated in the regulation of TO duplication of this microorganism is completely unknown.

Herein, we describe the isolation of a *M. genitalium* mutant showing an increased rate of TO duplication. The analyses performed reveal that the low levels of P110 and P140 present in this mutant are in the origin of the phenotype observed. Thus, our results indicate that P110 and P140 proteins are negative effectors of TO duplication in *M. genitalium*.

## Results

### Identification of a *M. genitalium* mutant with a transposon insertion in the *mg192* coding region

**Figure 1:**
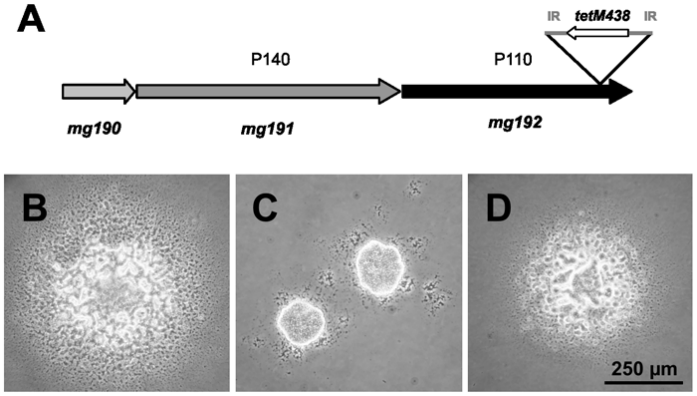
Altered colony morphology of the T192 mutant. (A) Scheme showing the insertion of the MTn*TetM438* minitransposon in the *mg192* coding region of the T192 mutant. Colony morphology of cells from the WT strain (B), the T192 mutant (C) and the T192C mutant (D) developed in soft agar. Cells from the WT strain and the complemented T192 mutant develop colonies with a disperse morphology while the T192 cells form colonies with a compact morphology indicative of motility deficiencies. IR, inverted repeat.

**Figure 2:**
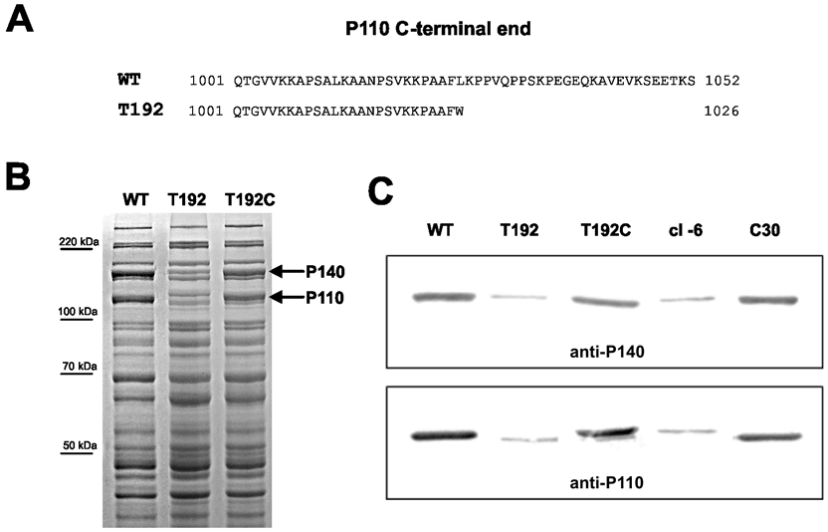
Analysis of P110 and P140 proteins. (A) Amino acid sequence of the P110 C-terminal end in the WT strain and the T192 mutant. (B) Protein profile of the WT strain and the mutants T192 and T192C by SDS-PAGE. (C) Western blotting analysis of the WT strain and the mutants T192, T192C, cI-6 and C30 using antiserum to P140 and P110.

Recently, we described a method to obtain *M. genitalium* mutants with an altered locomotion [13], [17]. By means of this procedure, a mutant with a minitransposon inserted in position 228984 of the *M. genitalium* genome and exhibiting a compact colony morphology (Fig. 1A–C) was isolated. In this mutant, designated T192, the transposon insertion was found in antisense near the 3′ end of the *mg192* gene that encodes for P110, a cytadherence-associated protein required for the stability of P140, the major cytadhesin of *M. genitalium* [24], [26]. Disruption of *mg192* in the T192 mutant was expected to generate a truncated protein 26 amino acids shorter than full-length P110 (Fig. 2A). SDS-PAGE and Western blotting using P110 antiserum confirmed the expression of a truncated P110 form but also revealed the presence of greatly reduced levels of that protein (Fig. 2B and C). As expected, P140 levels were found to be greatly reduced, confirming that both proteins are maintained at stoichiometric levels in *M. genitalium* [24]. In addition, a new band of about 115 kDa absent in the WT strain was observed in the protein profile of the T192 mutant. This band was previously observed in *M. genitalium* mutants lacking P110 [24] and its origin is still unknown.

### Cytadherence and motility of T192 mutant

**Figure 3:**
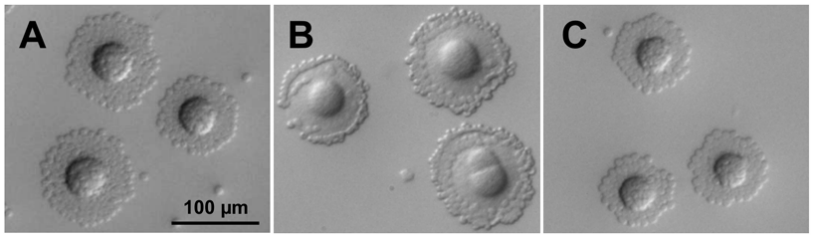
Hemadsorption activity analysis. Qualitative hemadsorption assay of the WT strain (A), the T192 mutant (B) and the T192C mutant (C). Colonies of the WT strain and the T192C mutant are fully covered by erythrocytes. In contrast, only the periphery of the T192 mutant colonies has erythrocytes bound.

**Figure 4:**
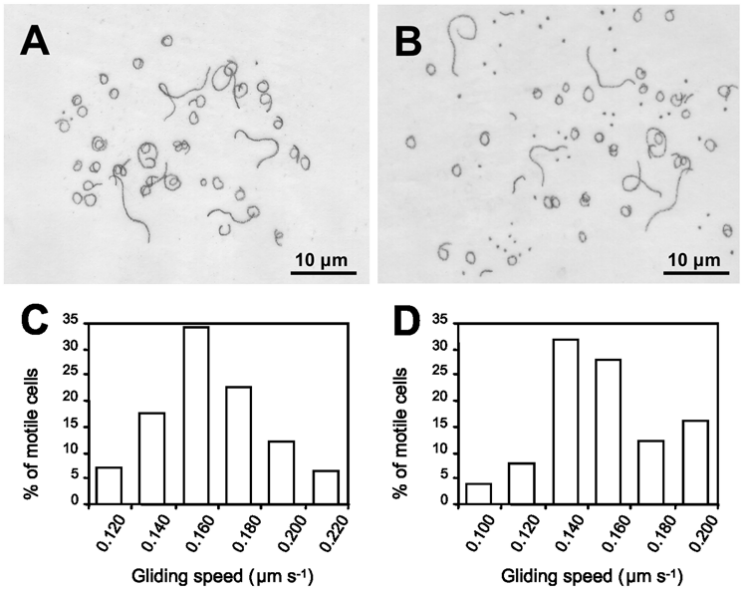
Gliding motility analysis. Tracks described by cells from the WT strain (A) and the T192 mutant (B) in two minutes. Distribution of the gliding velocities in the WT strain (C) and the T192 mutant (D). Note the presence of a large number of non-motile cells (scattered dots) in the population of the T192 mutant.

Consistent with the low levels of P110 and P140 observed, we found that the T192 mutant exhibited a capability to bind erythrocytes lower than that of the WT strain (Fig. 3A and B). As expected, the non-adherent mutant Δmg191 [24], used as a negative control, did not bind erythrocytes (data not shown). Motility of T192 cells was also analyzed by phase contrast microscopy (Fig. 4). The percentage of motile cells observed in the mutant T192 (45.6%) was clearly lower than the observed for the WT strain (93.8%). Surprisingly, motile cells from the T192 mutant showed a mean gliding speed (0.142±0.006 µm s^−1^) comparable to that observed for the WT cells (0.152±0.007 µm s^−1^). This finding is noticeable because all mycoplasma mutants deficient in gliding motility isolated previously exhibit both a lower number of motile cells and a reduced gliding velocity [13], [17], [23], [27], [28].

### Electron microscopy analyses of T192 mutant

**Figure 5:**
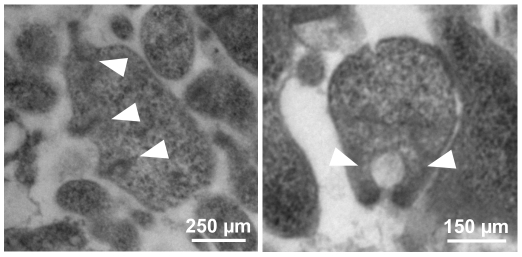
Transmission electron microscopy analysis of the T192 mutant. Arrowheads highlight the presence of several electron-dense cores inside a single cell.

**Figure 6:**
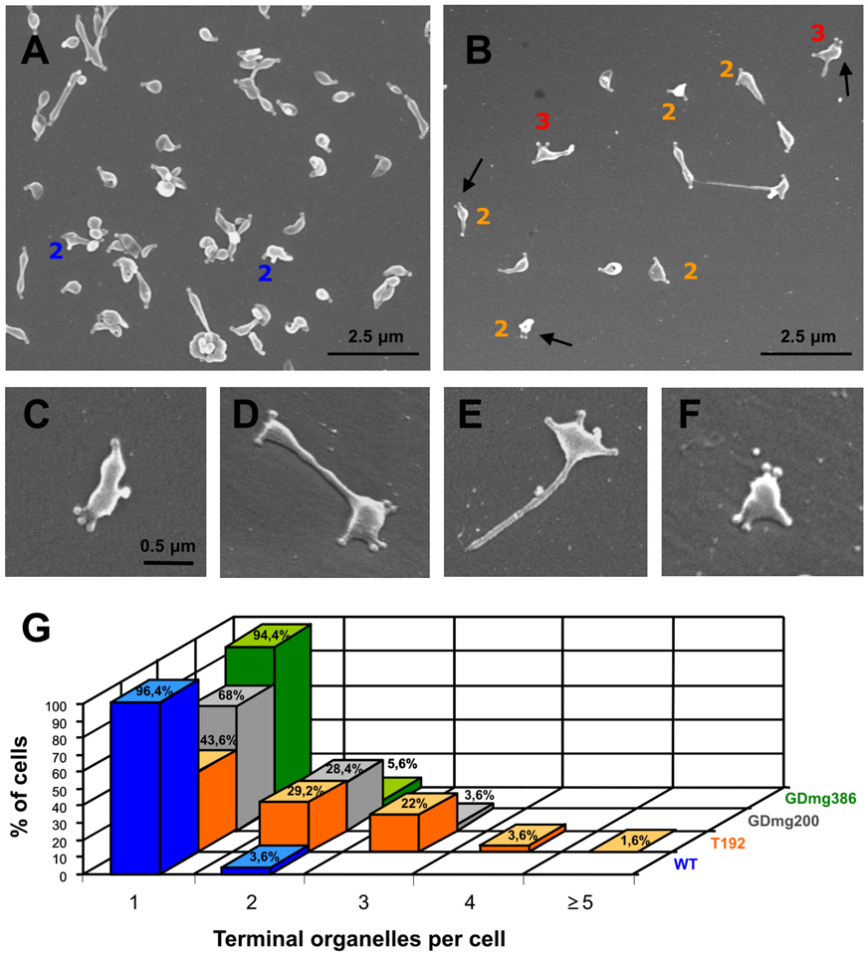
Scanning electron microscopy analysis. Electron micrographs of the WT strain (A) and the T192 mutant (B–F). In (G), graphic showing the percentage of cells with one, two, three, four and five or more TO in the WT strain, the T192 mutant, and the motility mutants GDmg200 c12 and GDmg386 c1. Arrows point out cells with two TO together at the same cell pole. The number of TO per cell are indicated in cells with more than one TO. Images in panels C to F are shown at the same magnification.

TO ultrastructure in the T192 mutant was analyzed by transmission electron microscopy (Fig. 5). The electron-dense cores observed were undistinguishable from those of the WT strain [13]. However, we clearly identified single cells showing two or more TOs with their corresponding electron-dense cores (Fig. 5, arrows). To obtain more accurate data about the number of TOs per cell, we used a standard procedure to visualize and quantify all the TO present in a single mycoplasma [6]. For this purpose, mycoplasmas were grown directly on a solid surface, fixed and examined by scanning electron microscopy (Fig. 6A–F). The percentage of cells with two TOs observed (29.2%; Fig. 6B and G) was markedly higher than that of the WT strain (3.6%; Fig. 6A and G). As reported previously [18], [19], cells with two TOs are probably dividing mycoplasmas since cells duplicate the tip structure just before the cytokinesis. In addition, we also observed a high proportion of cells with three TOs in the T192 mutant (22%; Fig. 6B and G), as well as the conspicuous presence of mycoplasmas with four (3.6%) and even five or more TOs per cell (1.6%; Fig. 6C–G). On the whole, a 27.2% of T192 cells showed multiple (three or more) terminal organelles (MTO). Such cells were never observed when examining the preparations of the WT strain indicating that the T192 mutant exhibits an increased rate of new TO formation.

### Complementation of T192 mutant with the *mg192* WT allele

The *mg192* WT allele was reintroduced into the T192 mutant by transposon delivery. For this purpose, cells from mutant T192 were electroporated in the presence of a minitransposon bearing the *mg192* gene under control of a *M. genitalium* constitutive promoter and the *aac(6′)-aph(2″)* marker which confers gentamicin resistance. Several transformants resistant to both gentamicin and tetracycline, and exhibiting a disperse colony morphology were isolated (Fig. 1D). SDS-PAGE and Western blotting analyses demonstrated the expression of full length P110 at nearly WT levels in the complemented mutants, designated T192C (Fig. 2B and C). As expected, a faint band corresponding to the truncated P110 protein was still detected in the T192C mutants (Fig. 2C). Further characterization of the complemented mutants demonstrated restoration of the hemadsorption activity (Fig. 3C) and the percentage of motile cells (89.2%). As expected, no cells exhibiting the MTO phenotype described above were detected in the T192C mutants (Table 1). Altogether, these results indicate that the transposon insertion in the *mg192* gene is in the origin of the MTO phenotype observed in the T192 mutant.

**Table 1:** Phenotypes of the analyzed strains.

Strain	P110/P140 levels*	Hemadsorption	% of motile cells	Mean gliding speed (µm s^−1^±SE)§	% of cells with MTO†
WT	++++	++++	93.8	0.152±0.007	0
T192	+	++	45.6	0.142±0.006	27.2
T192C	+++	++++	89.2	0.151±0.007	0
GDmg200	++++	+++	4.8	0.013±0.001	3.6
GDmg386	++++	+++	47.3	0.047±0.004	0
cI-6	+	++	42.5	0.140±0.004	20.8
C30	+++	++++	92.8	0.152±0.007	0

* Qualitative levels are indicated as: +, minimum; ++, intermediate-low; +++, intermediate-high; ++++, maximum.

§ SE, Standard error.

† MTO, Multiple (three or more) terminal organelles.

### Origin of the MTO phenotype in the T192 mutant

**Figure 7:**
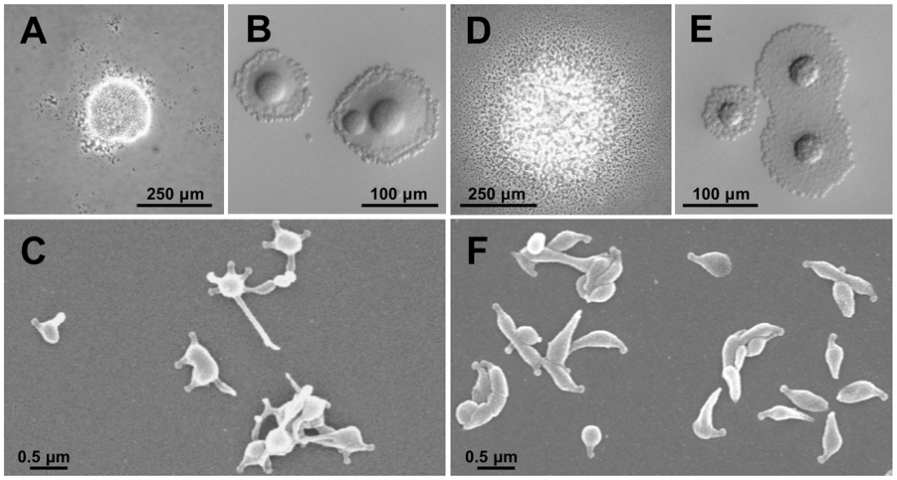
Phenotype of the cI-6 and c30 mutants. (A) Colony morphology, (B) qualitative hemadsorption activity and (C) scanning electron microscopy analysis of the cI-6 mutant. (D) Colony morphology, (E) qualitative hemadsorption activity and (F) scanning electron microscopy analysis of the C30 mutant.

At this point, we wondered whether the MTO phenotype was correlated with the low levels of P110 and P140 observed in the T192 mutant. To answer that question, a *M. genitalium* mutant showing levels of P110 and P140 similar to those observed in the T192 mutant was analyzed. This mutant was isolated in a previous work, when a WT copy of the P110 and P140 coding genes was reintroduced in a class I mutant by transposon delivery [24]. Originally, class I mutants have no P110 and extremely low levels of P140 [29]. The reintroduction of WT copies of P110 and P140 coding genes in those mutants generally restore the expression of these two proteins at WT levels. However, a few mutants showing low levels of expression of P110 and P140 were also isolated. One of these strains named cI-6 and showing greatly reduced levels of P110 and P140 (Fig. 2C) was analyzed and exhibited the same phenotype observed previously for the T192 mutant, including the high frequency of cells with MTOs (Table 1 and Fig. 7A, B and C). This result demonstrates that the MTO phenotype observed in the T192 mutant is a direct consequence of the low levels of P110 and P140.

**Figure 8:**
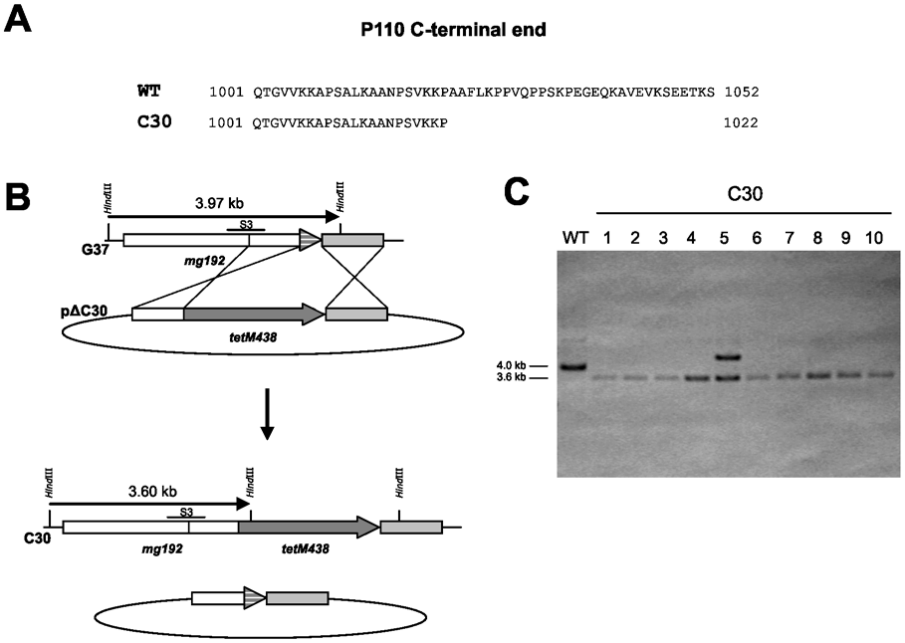
Construction of a *M. genitalium* mutant lacking the last 30 amino acids of P110. (A) Amino acid sequence of the P110 C-terminal end in the WT strain and the C30 mutant. (B) Schematic representation of the construction of the C30 mutant by homologous recombination using plasmid pΔC30. (C) Southern blot analysis of the C30 mutants using the fragment S3 as a probe. Detection of a single band of about 3.6 kb in nine of the transformants is consistent with the intended deletion in the *mg192* gene. Detection of two bands in the lane corresponding to clone 5 indicates the insertion of plasmid pΔC30 in the genome of this transformant by a single crossover. This mutant was not considered for further analysis.

To further confirm that the expression of a truncated P110 does not lead to a MTO phenotype, a *M. genitalium* mutant expressing a P110 derivative lacking the last 30 amino acids was obtained by homologous recombination (Fig. 8A). In this mutant, designated C30, the last 90 nucleotides of the *mg192* gene excluding the STOP codon were deleted and replaced by the *tetM438* selectable marker cloned in the same orientation as the *mg192* locus (Fig. 8B). To obtain the C30 mutant, the *M. genitalium* WT strain was electroporated in the presence of plasmid pΔC30 (Fig. 8B). Southern blot analysis of ten tetracycline resistant transformants demonstrated the intended deletion in the *mg192* gene (Fig. 8C). As expected Western blot analysis confirmed the expression of a truncated P110 protein in the C30 mutants (Fig. 2C). The levels of P110 and P140 detected in the C30 mutants were clearly higher than those observed previously for T192 suggesting that additional factors are needed to explain the dramatic reduction of the levels of these proteins in the T192 mutant. All the C30 mutants analyzed exhibited a WT phenotype (Fig. 7D, E and F; Table 1) demonstrating also that the MTO phenotype observed in the T192 mutant is a consequence of the expression of P110 and P140 at greatly reduced levels rather than a direct effect derived from the deletion of the P110 C-term.

### Electron microscopy analyses of gliding deficient mutants

Gliding motility is required to segregate the nascent TO prior to cytokinesis [20]. In consequence, migration of new TOs to the opposite cell pole is expected to be slower and should take a longer time in mutants with gliding motility deficiencies. Since one of the possible effects derived from this extended lapse of time could be the development of additional TOs, we looked for the existence of cells with MTOs in several gliding mutants isolated earlier in our laboratory [17]. For this purpose, the previously characterized gliding deficient mutants GDmg386 c1 (mean gliding speed of 0.047 µm s^−1^) and GDmg200 c12 (mean gliding speed of 0.013 µm s^−1^) were also analyzed by scanning electron microscopy. Interestingly, we found that the majority of cells from the GDmg386 c1 mutant exhibited a single TO (94.4%, Fig. 6G) though a slight increase in the percentage of cells with two TOs was also observed (5.6%, Fig. 6G). On the other hand, analysis of the GDmg200 c12 mutant revealed the presence of a high percentage of cells with two TOs (28.4%, Fig. 6G) as well as the existence of some cells with three TOs (3.6%, Fig. 6G). These results demonstrate that a reduced locomotion raises the percentage of cells displaying two TOs and confirm the implication of mycoplasma gliding in TO migration [20]. However, it was noticeable the absence of cells with MTOs in the GDmg386 mutant analyzed, in which the percentage of non-motile cells is comparable to that observed in the T192 mutant (52.7%). Similarly, despite the presence of a few cells with three TOs, the majority of cells from the GDmg200 mutant exhibited a single TO (68%), even when the percentage of non-motile cells in this mutant reaches the 95.2% of the population. In addition, no cells were seen exhibiting more than three terminal organelles. These observations indicate that the MTO phenotype displayed by the T192 mutant cannot be explained solely by a deficient locomotion and reveal that the increased frequency of TOs is a genuine trait of the T192 strain.

### DNA content of T192 and gliding deficient mutants

**Figure 9:**
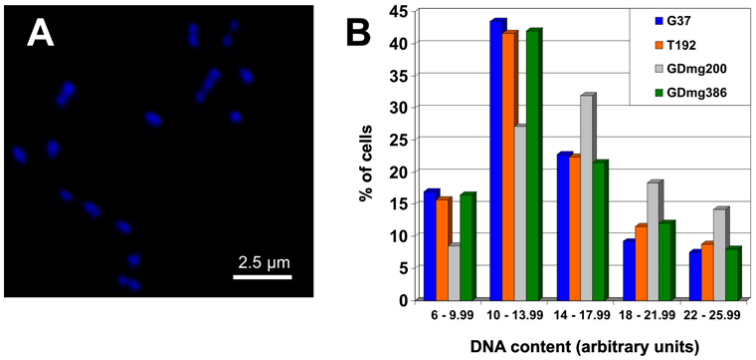
DNA content analysis. (A) Cells from the WT strain stained with DAPI. (B) Distribution of the DNA content per cell (DAPI mean density) of the WT strain, the T192 mutant, and the motility mutants GDmg200 c12 and GDmg386 c1.

Since the initiation of chromosome replication in mycoplasmas seems to be coincident with TO duplication [21], the presence of a higher rate of TO duplication in the T192 mutant prompted us to wonder about the DNA content of cells from that strain. We found that T192 cells had a DNA content comparable to that of WT cells (Fig. 9A and B), suggesting that the cells with MTOs do not experience a concomitant increase in the number of newly replicated chromosomes. In agreement with this result, T192 shows a growth rate similar to that of the WT strain (data not shown). In contrast, we observed a moderate increase in the number of cells with a high DNA content in the GDmg200 mutant (Fig. 9B). Probably, the slower gliding velocity of the cells from this mutant delays both separation of daughter cells and chromosome segregation increasing in this way the proportion of cells with a high DNA content. On the other hand, we did not find significant differences between the DNA content of the GDmg386 mutant analyzed and the WT strain (Fig. 9B), a result that is consistent with the moderate gliding deficiencies and the low percentage of cells exhibiting two TOs observed in this mutant.

## Discussion

The terminal organelle is a complex structure involved in many and diverse aspects of the biology of mycoplasmas such as cell adherence, motility or cell division. Despite the important functions carried out by this structure, most of them involved in mycoplasma virulence, little is known about the mechanisms regulating its duplication and how these mechanisms are interlaced with those regulating the cell cycle of mycoplasmas. In a previous report, we demonstrated the strict requirement of P110 and P140 cytadhesins for TO development [24]. Now, we show that low levels of P110 and P140 lead to an increased rate of new TO formation, indicating that these two proteins are also negative effectors of the duplication of this structure and revealing a role for these cytoskeletal proteins in *M. genitalium* cell division.

The T192 mutant described here was originally isolated in a large scale genetic screen devised to obtain mycoplasma gliding mutants and accordingly, 54.4% of the cells from this mutant are non-motile. However, gliding velocity of T192 the cells is not significantly different from that exhibited by the WT cells. These data indicate that the gliding apparatus of the T192 mutant is fully functional and suggest that the increased ratio of non-motile cells observed is not caused by defects in the gliding mechanics. In contrast, since mycoplasmas with nascent TOs are non-motile or barely motile [20], the high percentage of non-motile cells found in the T192 mutant is consistent with the increased rate of new TO formation. That is, the non-motile cells observed in the microcinematographies of the T192 mutant likely correspond to the mycoplasmas with nascent TOs identified by electron microscopy. Interestingly, many T192 cells exhibit a single TO (43.6%, Fig. 6) indicating that a considerable amount of daughter cells receive a single TO during the cell division process. This fact indicates that the segregation of cells with a single TO is favored even in cells with MTOs.

An additional question that arises from the inspection of the T192 mutant phenotype is the specific origin of the dramatic reduction of the P110 levels. We demonstrate that mutants expressing a truncated P110 lacking the last thirty amino acids and obtained by homologous recombination show levels of P110 and P140 closer to those exhibited by the WT strain. This result favors an alternative explanation to the low levels of P110 in the T192 mutant based on the genetic structure of the transposon insertion in this strain. Since the *tetM438* marker gene in the transposon insertion is in antisense with the *mg192* coding region, it is possible that transcripts run on the marker gene could partially block the P110 translation (Fig. 1A). Given the low number of terminator sequences found in the *M. genitalium* genome, antisense transcripts are common in this microorganism and its presence has been detected previously in a number of genes including those located inside the MgPa operon [30]. Work is in progress to test this hypothesis that could provide clues about the regulatory role of the antisense transcripts in *M. genitalium*.

Opposite to the observed in *M. pneumoniae* [20], we provide strong evidences that *M. genitalium* WT cells and cells with moderate gliding deficiencies do not duplicate the TO more than once prior to cytokinesis. These observations support the existence of a regulatory mechanism that prevents the overlapping of successive cell division cycles in *M. genitalium* and ensures the delivery of a single TO to each daughter cell. Among the possible benefits derived from the existence of such mechanism, it is clear that both daughter cells may be able to resume their gliding motion soon after the completion of cell division. Our results also provide additional evidences supporting that this regulatory mechanism involves P140 and P110 since, in the presence of low levels of both proteins, no deficiencies in the gliding mechanics are detected but a large number of cells develop MTOs. However, the observation of a few cells with three TOs in the GDmg200 mutant indicates that this regulatory mechanism can be occasionally overcome in mutants with severe gliding deficiencies.

Finally, TO duplication and DNA replication seem to be uncoupled in T192 cells. In this sense, despite the presence of cells with MTOs, the DNA content observed is not higher than in the WT cells, indicating that the synthesis of a new TO does not trigger a new round of DNA replication. This result raises the possibility that DNA replication is triggering the development of a new TO or well that both processes are triggered by a common effector in the WT strain. As observed in *Caulobacter crescentus* [31], the ubiquitous bacterial DNA replication initiator DnaA could play additional roles in *M. genitalium* triggering also TO duplication. Further investigation is needed to ascertain the check points and the specific function/s of P140 and P110 in the regulation of TO duplication. However, our finding regarding the role of these proteins as negative effectors of this process immediately suggests two attractive possibilities. On one hand, these proteins found in high levels elsewhere along the cell cycle may be sequestering any of the key components needed to assemble a new TO. On the other hand, a transient downregulation of P140 and P110 just before TO duplication, may allow the assembly of the new structure. Work is in progress to test both proposals.

## Materials and Methods

### Strains and primers used in this work

All the strains and primers used in this work are summarized in Tables 2 and 3.

**Table 2:** Strains used in this work.

Strain	Description
G37	*M. genitalium* WT strain
T192	G37 strain with a MTn*TetM438* minitransposon inserted in the *mg192* gene
T192C	T192 mutant complemented with plasmid pMTn*GmMG192*
GDmg200	G37 strain with a MTn*TetM438* minitransposon inserted in the *mg200* gene [17]
GDmg386	G37 strain with a MTn*TetM438* minitransposon inserted in the *mg386* gene [17]
cI-6	cI mutant complemented with plasmid pMTn*TetMG191-192* [24]
C30	G37 strain expressing a P110 C-terminal derivative lacking the last 30 amino acids

**Table 3:** Primers used in this work.

Primer	Sequence
Tc Up	5′-GGTAGTTTTTCCTGCATCAACATG
Tc Down	5′-CGTCGTCCAAATAGTCGGATAG
mg192Cter-5′	5′-GGGCCCTGGTAATGATTCATTGCTTTC
C30-3′	5′-GTCGACTTAAGGTTTTTTAACACTAGGATTAGC
DRmg192-5′	5′-GGATCCTTTTAACCTTTCAATAACCTAAAACAC
DRmg192-3′	5′-CTGCAGCACTATCCTTGTTAAATTGATCTC

### Construction of plasmids pΔC30 and pMTn*GmMG192*

To obtain plasmid pΔC30, a 1.1 kb fragment corresponding to the 3′ end of the *mg192* coding region (bases 1989 to 3069) was amplified by PCR using mg192Cter-5′ and C30-3′ primers (Table 2), cloned into an *Eco*RV digested pBE plasmid [32], and digested with *Apa*I and *Sal*I. Separately, a 1.3 kb fragment corresponding to the downstream region of *mg192* gene was amplified using DRmg192-5′ and DRmg192-3′ primers (Table 2), cloned into an *Eco*RV digested pBE plasmid, and digested with *Bam*HI and *Pst*I. Finally, both flanking regions and a *Sal*I-*Bam*HI digested *tetM438* marker were cloned into a *Apa*I-*Pst*I digested pBSK plasmid. To obtain plasmid pMTn*GmMG192*, the *mg192* WT allele with a promoter sequence was excised from plasmid pTn*TetMG192* (Burgos *et al*., 2006) using *Sal*I and cloned into a *Sal*I digested pMTn*Gm* vector [32].

### Culture conditions and transformation

*M. genitalium* cells were grown in SP-4 broth [33] at 37°C under a 5% CO_2_. For standard colony development, mycoplasmas were plated in SP-4 medium supplemented with 0.8% agar (Difco). Alternatively, gliding-dependent colony morphology was analyzed on cells previously attached to cell culture dishes (Corning) and covered with SP-4 broth containing 0.5% low melting point agarose (Iberlabo). To select for antibiotic resistant strains, SP-4 medium was supplemented with tetracycline 2 µg ml^−1^ (Roche), gentamicin 120 µg ml^−1^ (Sigma) or both. Transformation of *M. genitalium* was achieved by electroporation [32].

### DNA manipulations

Genomic DNA was isolated using E.Z.N.A. Bacterial DNA Kit (Omega Bio-tek). Sequencing of genomic DNA from the transposon mutants was performed with fluorescent dideoxynucleotides using Big Dye 3.0 Terminator Kit (Applied Biosystems) and Tc Up and Tc Down primers (Table 2), according to recommendations of the manufacturer. Southern blot hybridization was performed using the Dig DNA labeling and detection kit (Roche) and the S3 fragment as a probe [24].

### SDS-PAGE and Western blotting

The separation of mycoplasma proteins by SDS-PAGE followed standard procedures. Western blots using P110 polyclonal antibodies were performed as described previously [24].

### Hemadsorption activity and microcinematography

Qualitative hemadsorption activity was assessed as described previously [17]. Gliding motility data were obtained by microcinematography as described previously [17].

### Electron microscopy analysis

Transmission and scanning electron microscopy analyses were performed as described previously [13]. About 250 cells from each strain were analyzed to determine the percentage of cells with a particular number of TOs.

### Fluorescence microscopy analysis

*M. genitalium* cells passed through 0.45 µm filters (Nalgene) were grown on permanox chamber slides (Nunc) for 16 h. Then, SP-4 medium was removed, cells were washed with PBS buffer, fixed with 1% glutaraldehyde and stained with 50 µl of Vectashield Hardset mounting medium containing 4′,6-diamidino-2-phenylindole (DAPI) (Vector Laboratories). DAPI stained mycoplasmas were observed using an Axio Observer inverted research microscope from Carl Zeiss with AxioVision 4.6.1 software. All the images were acquired at identical exposure times (500 ms). The DAPI mean density of about 100 individual cells from each strain was measured with the Scion Image software using the Measure option from the Menu Analyze. For each field analyzed, a mean density of the background was averaged and subtracted from all the measurements taken.

## References

[1] Hegermann J, Herrmann R, Mayer F (2002). Cytoskeletal elements in the bacterium *Mycoplasma pneumoniae*.. Naturwissenschaften.

[2] Nakane D, Miyata M (2007). Cytoskeletal “jellyfish” structure of *Mycoplasma mobile*.. Proc Natl Acad Sci U S A.

[3] Rosengarten R, Kirchhoff H (1987). Gliding motility of *Mycoplasma* sp. nov. strain 163K.. J Bacteriol.

[4] Bredt W, Radestock U (1977). Gliding motility of *Mycoplasma pulmonis*.. J Bacteriol.

[5] Taylor-Robinson D, Bredt W (1983). Motility of *Mycoplasma* strain G37.. Yale J Biol Med.

[6] Hatchel JM, Balish MF (2008). Attachment organelle ultrastructure correlates with phylogeny, not gliding motility properties, in *Mycoplasma pneumoniae* relatives.. Microbiology.

[7] Hatchel JM, Balish RS, Duley ML, Balish MF (2006). Ultrastructure and gliding motility of *Mycoplasma amphoriforme*, a possible human respiratory pathogen.. Microbiology.

[8] Radestock U, Bredt W (1977). Motility of *Mycoplasma pneumoniae*.. J Bacteriol.

[9] Fraser CM, Gocayne JD, White O, Adams MD, Clayton RA (1995). The minimal gene complement of *Mycoplasma genitalium*.. Science.

[10] Herrmann R, Reiner B (1998). *Mycoplasma pneumoniae* and *Mycoplasma genitalium*: a comparison of two closely related bacterial species.. Curr Opin Microbiol.

[11] Regula JT, Boguth G, Gorg A, Hegermann J, Mayer F (2001). Defining the mycoplasma ‘cytoskeleton’: the protein composition of the Triton X-100 insoluble fraction of the bacterium *Mycoplasma pneumoniae* determined by 2-D gel electrophoresis and mass spectrometry.. Microbiology.

[12] Gobel U, Speth V, Bredt W (1981). Filamentous structures in adherent *Mycoplasma pneumoniae* cells treated with nonionic detergents.. J Cell Biol.

[13] Pich OQ, Burgos R, Ferrer-Navarro M, Querol E, Pinol J (2008). Role of *Mycoplasma genitalium* MG218 and MG317 cytoskeletal proteins in terminal organelle organization, gliding motility and cytadherence.. Microbiology.

[14] Meng KE, Pfister RM (1980). Intracellular structures of *Mycoplasma pneumoniae* revealed after membrane removal.. J Bacteriol.

[15] Hasselbring BM, Krause DC (2007). Cytoskeletal protein P41 is required to anchor the terminal organelle of the wall-less prokaryote *Mycoplasma pneumoniae*.. Mol Microbiol.

[16] Burgos R, Pich OQ, Querol E, Pinol J (2008). Deletion of the *Mycoplasma genitalium MG_217* gene modifies cell gliding behaviour by altering terminal organelle curvature.. Mol Microbiol.

[17] Pich OQ, Burgos R, Ferrer-Navarro M, Querol E, Pinol J (2006). *Mycoplasma genitalium mg200* and *mg386* genes are involved in gliding motility but not in cytadherence.. Mol Microbiol.

[18] Boatman ES, Barile MF, Razin S (1979). Morphology and ultrastructure of the *mycoplasmatales*.. The mycoplasmas, vol I.

[19] Bredt W (1968). Motility and multiplication of *Mycoplasma pneumoniae*. A phase contrast study.. Pathol Microbiol (Basel).

[20] Hasselbring BM, Jordan JL, Krause RW, Krause DC (2006). Terminal organelle development in the cell wall-less bacterium *Mycoplasma pneumoniae*.. Proc Natl Acad Sci U S A.

[21] Seto S, Layh-Schmitt G, Kenri T, Miyata M (2001). Visualization of the attachment organelle and cytadherence proteins of *Mycoplasma pneumoniae* by immunofluorescence microscopy.. J Bacteriol.

[22] Quinlan DC, Maniloff J (1972). Membrane association of the deoxyribonucleic acid growing-point region in *Mycoplasma gallisepticum*.. J Bacteriol.

[23] Burgos R, Pich OQ, Querol E, Pinol J (2007). Functional analysis of the *Mycoplasma genitalium* MG312 protein reveals a specific requirement of the MG312 N-terminal domain for gliding motility.. J Bacteriol.

[24] Burgos R, Pich OQ, Ferrer-Navarro M, Baseman JB, Querol E (2006). *Mycoplasma genitalium* P140 and P110 cytadhesins are reciprocally stabilized and required for cell adhesion and terminal-organelle development.. J Bacteriol.

[25] Hasselbring BM, Krause DC (2007). Proteins P24 and P41 function in the regulation of terminal-organelle development and gliding motility in *Mycoplasma pneumoniae*.. J Bacteriol.

[26] Hu PC, Schaper U, Collier AM, Clyde WA, Horikawa M (1987). A *Mycoplasma genitalium* protein resembling the *Mycoplasma pneumoniae* attachment protein.. Infect Immun.

[27] Miyata M, Yamamoto H, Shimizu T, Uenoyama A, Citti C (2000). Gliding mutants of *Mycoplasma mobile*: relationships between motility and cell morphology, cell adhesion and microcolony formation.. Microbiology.

[28] Hasselbring BM, Page CA, Sheppard ES, Krause DC (2006). Transposon mutagenesis identifies genes associated with *Mycoplasma pneumoniae* gliding motility.. J Bacteriol.

[29] Mernaugh GR, Dallo SF, Holt SC, Baseman JB (1993). Properties of adhering and nonadhering populations of *Mycoplasma genitalium*.. Clin Infect Dis.

[30] Lluch-Senar M, Vallmitjana M, Querol E, Pinol J (2007). A new promoterless reporter vector reveals antisense transcription in *Mycoplasma genitalium*.. Microbiology.

[31] Hottes AK, Shapiro L, McAdams HH (2005). DnaA coordinates replication initiation and cell cycle transcription in *Caulobacter crescentus*.. Mol Microbiol.

[32] Pich OQ, Burgos R, Planell R, Querol E, Pinol J (2006). Comparative analysis of antibiotic resistance gene markers in *Mycoplasma genitalium*: application to studies of the minimal gene complement.. Microbiology.

[33] Tully JG, Rose DL, Whitcomb RF, Wenzel RP (1979). Enhanced isolation of *Mycoplasma pneumoniae* from throat washings with a newly-modified culture medium.. J Infect Dis.

